# Supplementary figures and images for: Ubiquitin ligase activity inhibits Cdk5 to control axon termination
Source: PLoS Genet. 2022 Apr 14;18(4):e1010152. doi: 10.1371/journal.pgen.1010152 (PMC9041834; doi:10.1371/journal.pgen.1010152)

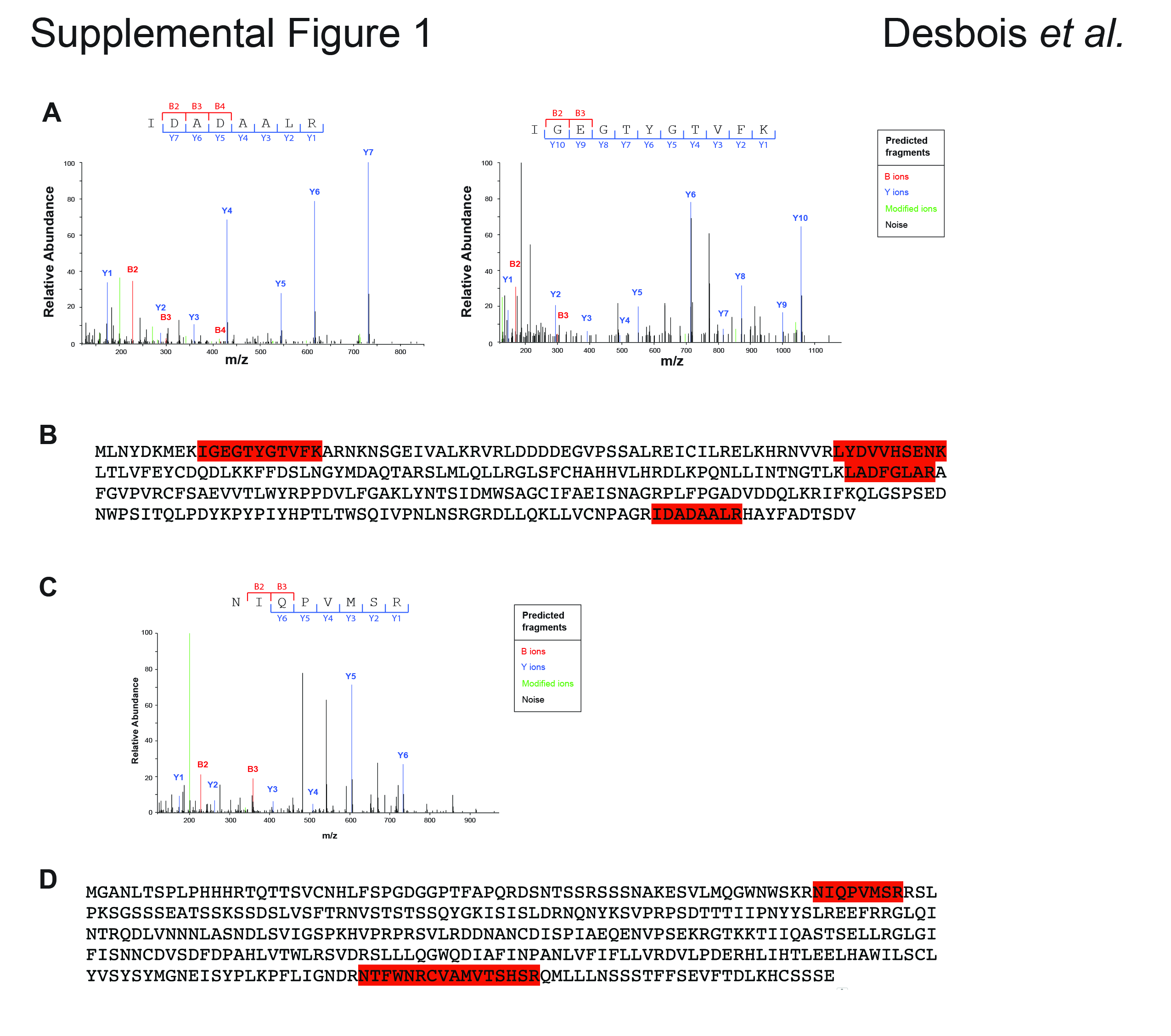

Supplement: S1 Fig — A) LC MS-MS spectra for two CDK-5 peptides identified in GS::RPM-1 LD samples. B) Highlighted in CDK-5 sequence are four unique CDK-5 peptides (red) identified in GS::RPM-1 LD substrate ‘trap’ samples. C) LC MS-MS spectrum of one CDKA-1 peptide identified in GS::RPM-1 LD samples. D) Highlighted in CDKA-1 sequence are two unique peptides (red) identified in GS::RPM-1 LD samples. (TIF) [file pgen.1010152.s001.tif]

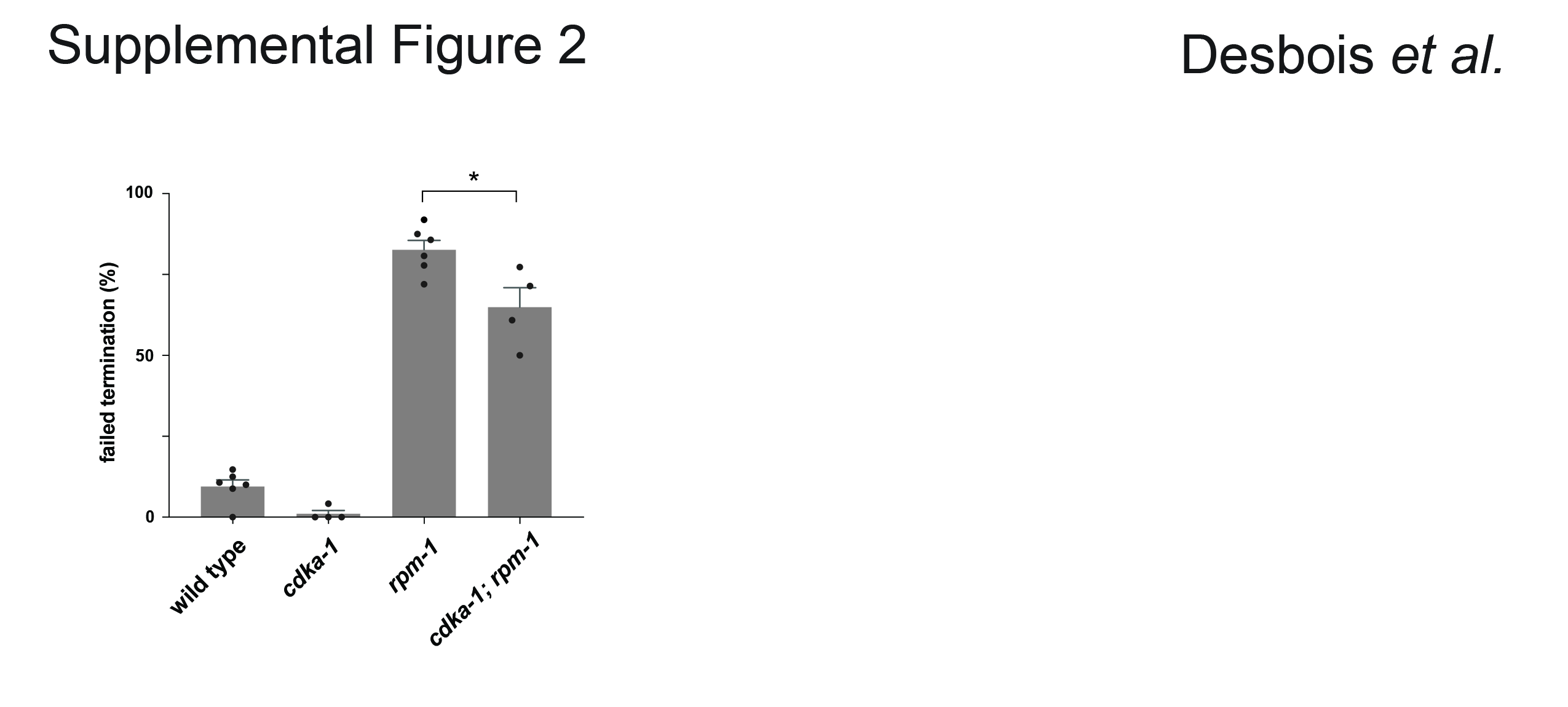

Supplement: S2 Fig — Quantitation indicates ALM axon termination defects are reduced in cdka-1; rpm-1 double mutants compared to rpm-1 single mutants. Means (bars) are shown for 4 or more counts (black dots, 20 or more worms/count) for each genotype. Error bars indicate SEM. Significance assessed using Student’s t-test. * p<0.05. (TIF) [file pgen.1010152.s002.tif]

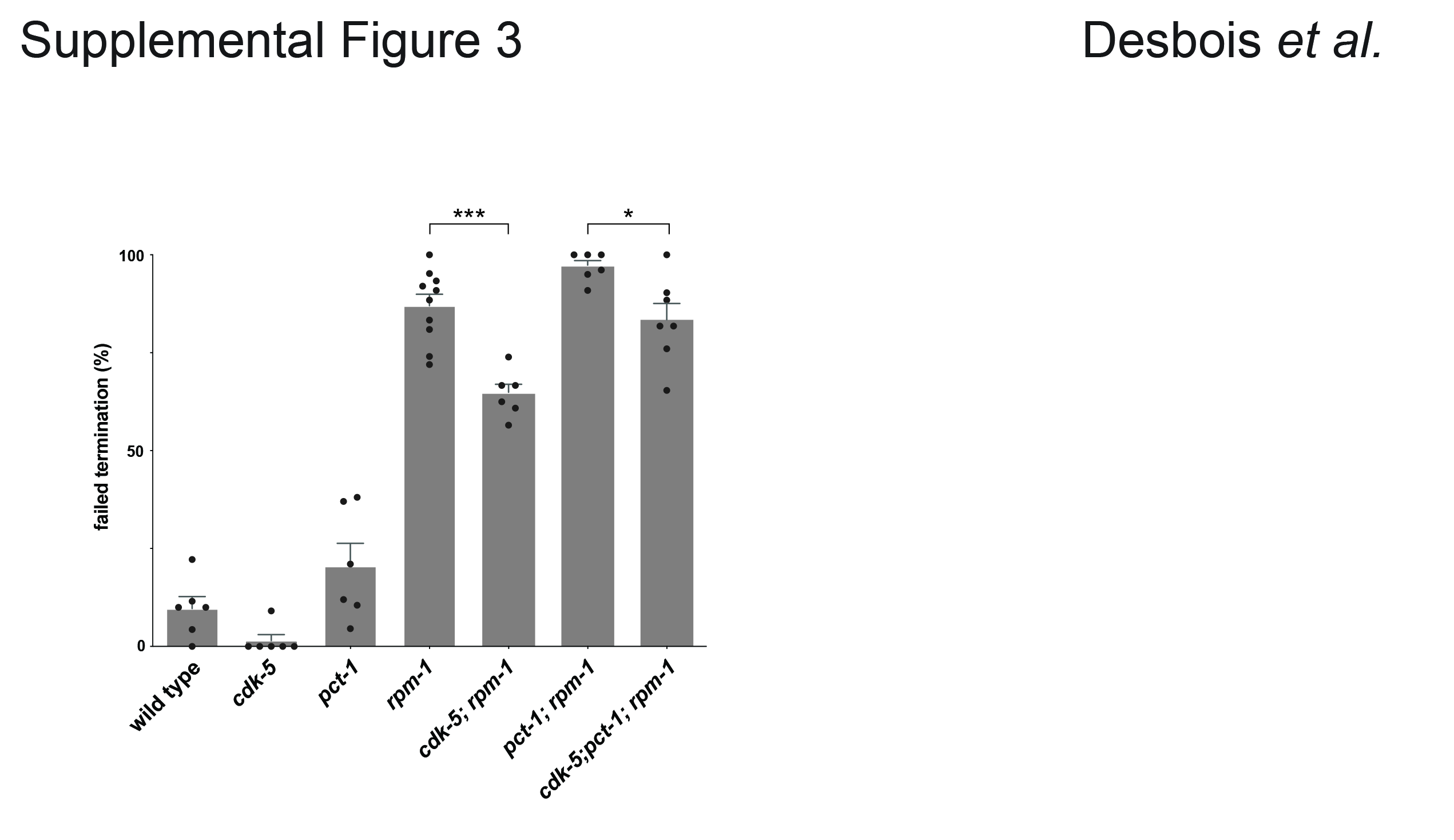

Supplement: S3 Fig — Quantitation of axon termination defects in ALM neurons for indicated genotypes. Reduced frequency of axon termination defects occurs in cdk-5; rpm-1 double mutants, but not pct-1; rpm-1 double mutants. Suppression of termination defects is not increased in cdk-5; pct-1; rpm-1 triple mutants compared to cdk-5; rpm-1 double mutants. Results suggest that CDK-5 and PCT-1 do not function redundantly during axon termination. Means (bars) are shown for 6 or more counts (black dots, 20 or more worms/count) for each genotype. Error bars indicate SEM. Significance assessed using Student’s t-test. * p<0.05, ** p<0.01. (TIF) [file pgen.1010152.s003.tif]

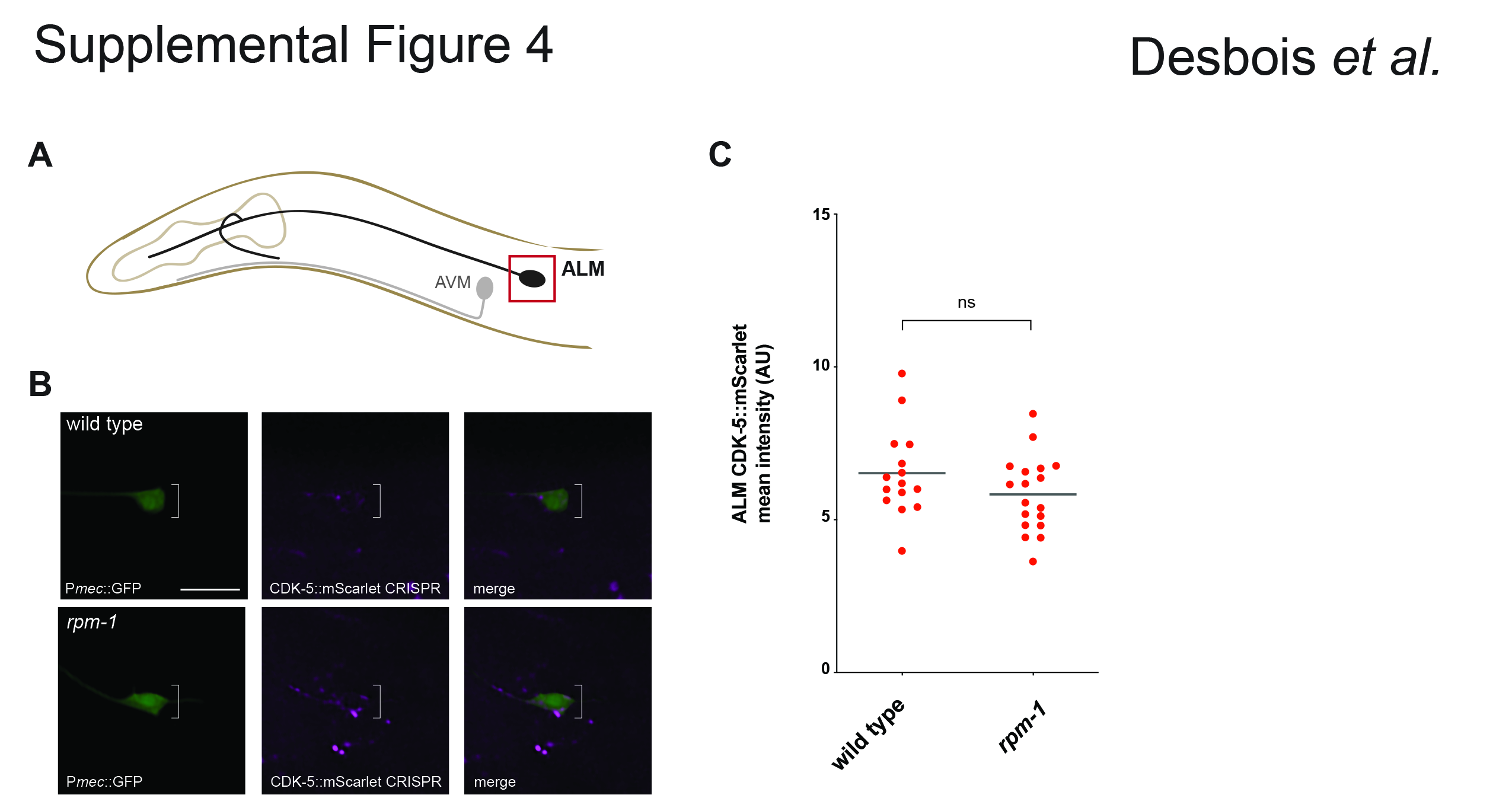

Supplement: S4 Fig — A) Schematic shows region of ALM mechanosensory neurons imaged (red box). B) Representative images of ALM soma from wildtype and rpm-1 (lf) mutants. Shown is transgenic GFP expressed in mechanosensory neurons (PmecGFP; left, green), CDK-5::mScarlet CRISPR (middle, magenta) and merged image (right). C) Quantitation shows CDK-5::mScarlet levels in ALM soma are not different between wildtype animals and rpm-1 (lf) mutants. Means (grey lines) are shown for each genotype with individual data points (red dots) representing single animals. Significance assessed using Student’s t-test. ns, non-significant. (TIF) [file pgen.1010152.s004.tif]

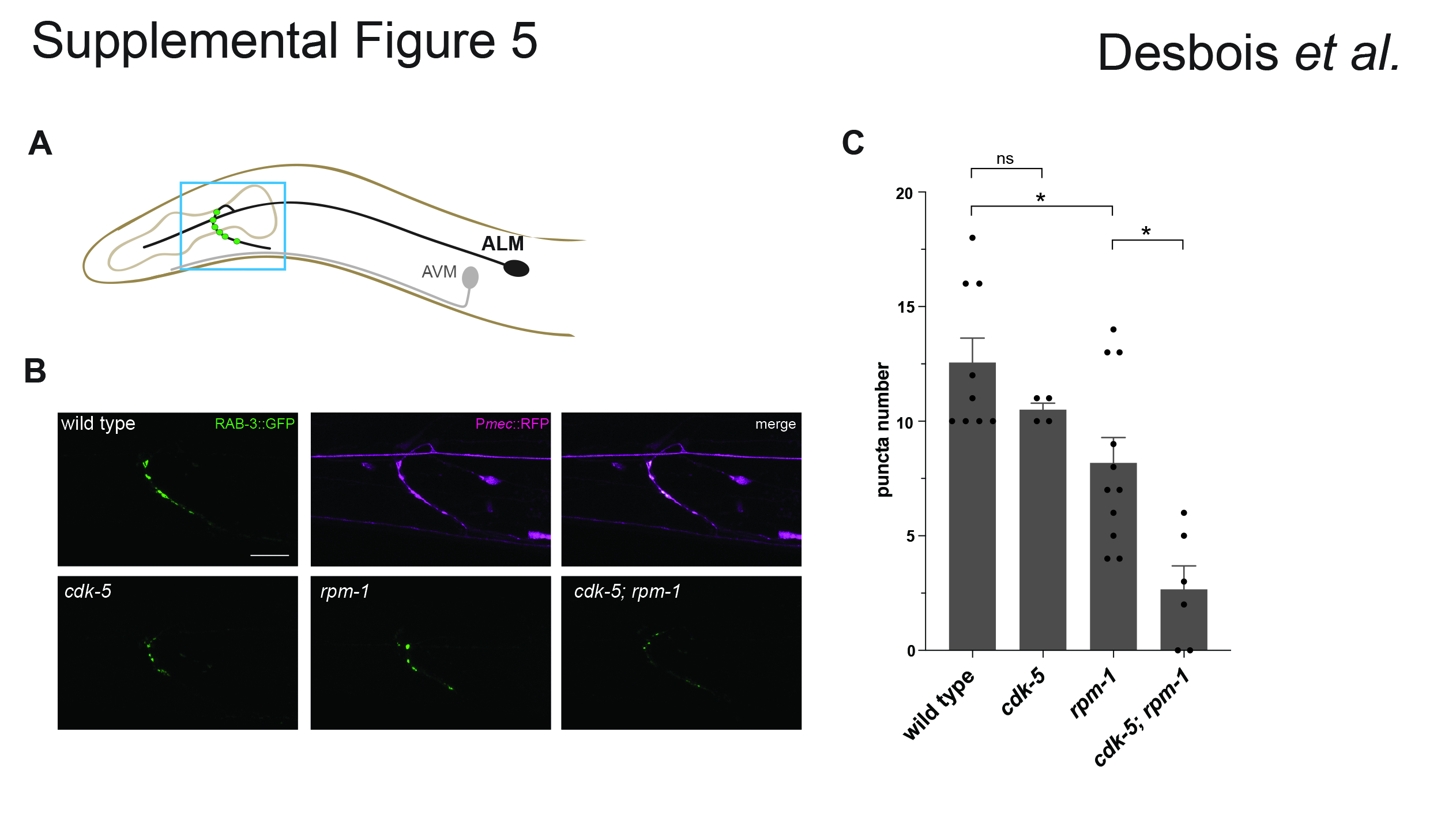

Supplement: S5 Fig — RPM-1 and CDK-5 function in parallel to regulate synapse formation in ALM mechanosensory neurons. A) Schematic shows primary axon and collateral synaptic branch of ALM mechanosensory neurons. Highlighted in green on collateral branch are presynaptic sites labeled by RAB-3::GFP. B) Shown are representative confocal images of presynaptic RAB-3::GFP puncta for indicated genotypes. For wildtype, images are shown for GFP::RAB-3 (upper left, green), RFP expressed in mechanosensory neurons (PmecRFP; upper middle, magenta) and merged image (upper right). C) Quantitation of GFP::RAB-3 puncta in ALM neurons for indicated genotypes. Note rpm-1 mutants have reduced numbers of RAB-3 puncta, and these defects are enhanced in cdk-5; rpm-1 double mutants. Means (bars) are shown for each genotype (black dots indicate individual worms). Error bars indicate SEM. Significance assessed using Student’s t-test with Bonferroni correction. ns, non-significant; * p<0.05. Scale bar 10 μm. (TIF) [file pgen.1010152.s005.tif]
