# Supplementary material for: Ubiquitin ligase activity inhibits Cdk5 to control axon termination
Source: PLoS Genet. 2022 Apr 14;18(4):e1010152. doi: 10.1371/journal.pgen.1010152 (PMC9041834; doi:10.1371/journal.pgen.1010152)
Supplement: S1 Table — (DOCX) [file pgen.1010152.s006.docx]

**Ubiquitin ligase activity inhibits Cdk5 to control axon termination**

Desbois *et al.*

**S1 Table Transgenic and CRISPR Strains**

| **Figure** | **Strain name** | **Genotype** |
| --- | --- | --- |
| Figure 1 | XMN829 | *muIs32* II; *rpm-1*(*ju44*) *bggIs9* [P_rpm-1_::GS::RPM-1; P_myo-2_:: mCherry; pha-1(+)] V |
| Figure 1 | XMN830 | *muIs32 bggIs19* [P_rpm-1_::GS::RPM-1 LD; P_myo-2_::mCherry; pha-1(+)] II; *rpm-1*(*ju44*) V |
| Figure 1 | XMN831 | *muIs32* II; *rpm-1*(*ju44*) V; *bggIs23* [P_rpm-1_::GS::GFP; P_myo-2_:: mCherry, pha-1(+)] |
| Figure 2 B | XMN841 | *rpm-1*(*bgg6* [GFP::RPM-1 CRISPR]) V |
| Figure 2 B, H, I | XMN948 | *rpm-1*(*bgg6 bgg40* [GFP::RPM-1 LD CRISPR]) V. |
| Figure 2 B, C, E, F, H, I | XMN1135 | *cdk-5*(*bgg52* [CDK-5::3xFLAG CRISPR]) III |
| Figure 2 B | XMN1275 | *cdk-5*(*bgg52*) III; *rpm-1*(*bgg6*) V |
| Figure 2 B, H, I | XMN1276 | *cdk-5*(*bgg52*) III; *rpm-1*(*bgg6 bgg40)* V. |
| Figure 2 C, E, F | XMN1133 | *fsn-1*(*bgg47* [GFP::FSN-1 CRISPR]) III |
| Figure 2 C, E, F | XMN1276 | *fsn-1*(*bgg47*) *cdk-5*(*bgg52*) III |
| Figure 2 E, F | XMN1254 | *fsn-1*(*bgg47*) *cdk-5*(*bgg52*) III; *rpm-1*(*bgg74* [*rpm-1* LD CRISPR]) V |
| Figure 2 H, I | XMN1306 | *fsn-1(gk429) cdk-5(bgg52)* III*; rpm-1(bgg6 bgg40)* V |
| Figure 3 B, E | XMN1176 | *muIs32* II; *rpm-1*(*bgg74* [*rpm-1* LD CRISPR]) V |
| Figure 3 B, E | XMN1315 | *muIs32* II; *cdk-5(ok626)* III*; rpm-1*(*bgg74*) V |
| Figure 4 B | 5 lines | *muIs32* II; *cdk-5*(*ok626*) III; *rpm-1*(*ju44*) V; *bggEx (P_cdk-5_::cdk-5 genomic*) |
| Figure 4 B, 5C | 7 lines | *muIs32* II; *cdk-5*(*ok626*) III; *rpm-1*(*ju44*) V; *bggEx* (*P_mec-3_::cdk-5 cDNA*) |
| Figure 4 C, D | 9 lines | *muIs32* II; *bggEx* (P_rgef-1_::cdk-5 cDNA) |
| Figure 4 C, D | 7 lines | *muIs32* II; *bggEx* (P_mec-3_::cdka-1 genomic) |
| Figure 4 C, D | 11 lines | *muIs32* II; *bggEx* (P_rgef-1_::cdk-5 cDNA; P_mec-3_::cdka-1 genomic) |
| Figure 4 D | 9 lines | *muIs32* II; *bggEx* (P_rgef-1_::mCherry PCR) |
| Figure 4 D | 10 lines | *muIs32* II; *bggEx* (P_mec-3_::mCherry PCR) |
| Figure 5 B | XMN1277 | *muIs32* II; *cdk-5*(*bgg71* [K33T CRISPR] *bgg77* [D144N CRISPR]) III |
| Figure 5 B | XMN1278 | *muIs32* II; *cdk-5*(*bgg71* *bgg77)* III; *rpm-1*(*ju44*) V |
| Figure 5 C | 3 lines | *muIs32* II; *cdk-5*(*ok626*) III; *rpm-1*(*ju44*) V; *bggEx* (*P_mec-3_::cdk-5 K33T D144N cDNA*) |
| Figure 6 and S4 | XMN1339 | *muIs32* II; *cdk-5(bgg57* [CDK-5::wrmScarlet CRISPR]*)* III |
| Figure 6 | 2 lines | *cdk-5(bgg57)* III*; bggEx (P_mec-3_::GFP::rpm-1 LD)* |
