# Supplementary material for: Ubiquitin ligase activity inhibits Cdk5 to control axon termination
Source: PLoS Genet. 2022 Apr 14;18(4):e1010152. doi: 10.1371/journal.pgen.1010152 (PMC9041834; doi:10.1371/journal.pgen.1010152)
Supplement: S2 Table — (DOCX) [file pgen.1010152.s007.docx]

**Ubiquitin ligase activity inhibits Cdk5 to control axon termination**

Desbois *et al.*

**S2 Table Injection Conditions**

| **Figure** | **Transgene** | **Injected Strain** | **Injection Mix** |
| --- | --- | --- | --- |
| Figure 2 | CDK-5::3xFLAG   CRISPR  (*bgg52)* | N2 | 5 μL tracrRNA (4 μg/μl) |
|  |  |  | 0.4 μL dpy-10 crRNA (8 μg/μl) |
|  |  |  | 0.55 μL dpy-10 ssODN (500 ng/μl) |
|  |  |  | 1.0 μL CDK-5::3xFLAG crRNA (8 μg/μl) |
|  |  |  | 2.2 or 3.3 μL CDK-5::3xFLAG ssODN repair template (1 or 1.5 μg/μl) |
|  |  |  | 0.5 μL KCl (1M) |
|  |  |  | 0.75 μL Hepes pH7.4 (200mM) |
|  |  |  | 4.6 or 5.7 μL dH2O |
|  |  |  | 5 μL Cas9 (10 μg/μl) |
| Figure 2 | GFP::FSN-1  CRISPR  (*bgg47)* | N2 | 5 μL tracrRNA (4 μg/μl) |
|  |  |  | 0.4 μL dpy-10 crRNA (8 μg/μl) |
|  |  |  | 0.55 μL dpy-10 ssODN (500 ng/μl) |
|  |  |  | 1.0 μL GFP::FSN-1 crRNA (8 μg/μl) |
|  |  |  | 8.5 μL GFP::FSN-1 PCR repair template (609 ng/μl) |
|  |  |  | 0.5 μL KCl (1M) |
|  |  |  | 0.75 μL Hepes pH7.4 (200mM) |
|  |  |  | 5 μL Cas9 (10 ug/μl) |
|  |  |  | 5 μL tracrRNA (4 μg/μl) |
| Figure 2  Figure 3 | RPM-1 LD CRISPR  (*bgg74)* | N2 | 5 μL tracrRNA (4 μg/μl) |
|  |  |  | 0.4 μL dpy-10 crRNA (8 μg/μl) |
|  |  |  | 0.55 μL dpy-10 ssODN (500 ng/μl) |
|  |  |  | 1.0 μL RPM-1 LD crRNA (8 μg/μl) |
|  |  |  | 2.2 μL RPM-1 LD ssODN repair template (1 μg/μl) |
|  |  |  | 0.5 μL KCl (1M) |
|  |  |  | 0.75 μL Hepes pH7.4 (200mM) |
|  |  |  | 4.6μL dH2O |
| Figure 4 | CDK-5 mechanosensory neuron rescue | *muIs32;*  *cdk-5 (ok626);*  *rpm-1(ju44)* | 10ng/μL P_mec-3_::cdk-5 cDNA (pBG-GY943) |
|  |  |  | 50ng/μL P_ttx-3_::RFP (pBG-41) |
|  |  |  | 10ng/μL P_rps-27_::NeoR (pBG-264) |
|  |  |  | 30ng/μL pBluescript (pBG-49) |
| Figure 4 | CDK-5 rescue | *muIs32;*  *cdk-5 (ok626);*  *rpm-1(ju44)* | 50ng/μL P_cdk-5_::cdk-5 genomic:: 3'UTR cdk-5 (pBG-354) |
|  |  |  | 50ng/μL P_ttx-3_::RFP (pBG-41) |
|  |  |  | 10ng/μL P_rps-27_::NeoR (pBG-264) |
| Figure 4 | Promoter control overexpression | *muIs32* | 5ng/μL P_rgef-1_::mcherry cDNA PCR |
|  |  |  | 10ng/μL P_rps-27_::NeoR (pBG-264) |
|  |  |  | 85ng/μL pBluescript (pBG-49) |
| Figure 4 | Promoter control overexpression | *muIs32* | 5ng/μL P_mec-3_::mcherry cDNA PCR |
|  |  |  | 10ng/μL P_rps-27_::NeoR (pBG-264) |
|  |  |  | 85ng/μL pBluescript (pBG-49) |
| Figure 4 | CDK-5 overexpression | *muIs32* | 5ng/μL P_rgef-1_::cdk-5 cDNA PCR |
|  |  |  | 10ng/μL P_rps-27_::NeoR (pBG-264) |
|  |  |  | 85ng/μL pBluescript (pBG-49) |
| Figure 4 | CDKA-1 overexpression | *muIs32* | 5ng/μL P_mec-3_::cdka-1 cDNA PCR |
|  |  |  | 10ng/μL P_rps-27_::NeoR (pBG-264) |
|  |  |  | 85ng/μL pBluescript (pBG-49) |
| Figure 4 | CDK-5 and CDKA-1 overexpression | *muIs32* | 5ng/μL P_rgef-1_::cdk-5 cDNA PCR |
|  |  |  | 5ng/μL P_mec-3_::cdka-1 cDNA PCR |
|  |  |  | 10ng/μL P_rps-27_::NeoR (pBG-264) |
|  |  |  | 80ng/μL pBluescript (pBG-49) |
| Figure 5 | *cdk-5 KD (bgg71[K33T] CRISPR)* | N2 | 5 μL tracrRNA (4 μg/μl) |
|  |  |  | 0.4 μL dpy-10 crRNA (8 μg/μl) |
|  |  |  | 0.55 μL dpy-10 ssODN (500 ng/μl) |
|  |  |  | 1.0 μL CDK-5 [K33T] crRNA (8 μg/μl) |
|  |  |  | 2.2 μL CDK-5 [K33T] ssODN repair template (1 μg/μl) |
|  |  |  | 0.5 μL KCl (1M) |
|  |  |  | 0.75 μL Hepes pH7.4 (200mM) |
|  |  |  | 4.6μL dH2O |
|  |  |  | 5 μL Cas9 (10 μg/μl) |
| Figure 5 | *cdk-5 KD (bgg77[D144N] CRISPR)* | XMN1150  *cdk-5 (bgg71)* | 5 μL tracrRNA (4 μg/μl) |
|  |  |  | 0.4 μL dpy-10 crRNA (8 μg/μl) |
|  |  |  | 0.55 μL dpy-10 ssODN (500 ng/μl) |
|  |  |  | 1.0 μL CDK-5 [D144N] crRNA (8 μg/μl) |
|  |  |  | 2.2 μL CDK-5 [D144N] ssODN repair template (1 μg/μl) |
|  |  |  | 0.5 μL KCl (1M) |
|  |  |  | 0.75 μL Hepes pH7.4 (200mM) |
|  |  |  | 4.6μL dH2O |
|  |  |  | 5 μL Cas9 (10 μg/μl) |
| Figure 6 | CDK-5::mScarlet  *(bgg57 CRISPR)* | N2 | 5 μL tracrRNA (4 μg/μl) |
|  |  |  | 0.4 μL dpy-10 crRNA (8 μg/μl) |
|  |  |  | 0.55 μL dpy-10 ssODN (500 ng/μl) |
|  |  |  | 1.0 μL CDK-5::wrmScarlet crRNA (8 μg/μl) |
|  |  |  | 6.8 μL CDK-5::wrmScarlet PCR repair template (1027 ng/μl) |
|  |  |  | 0.5 μL KCl (1M) |
|  |  |  | 0.75 μL Hepes pH7.4 (200mM) |
|  |  |  | 5 μL Cas9 (10 ug/μl) |
| Figure 6 | GFP::RPM-1 LD | *bgg57* | 20ng/μL P_mec-3_::GFP::RPM-1 LD |
|  |  |  | 10ng/μL P_rps-27_::NeoR (pBG-264) |
|  |  |  | 90ng/μL pBluescript (pBG-49) |
