# Supplementary material for: Ubiquitin ligase activity inhibits Cdk5 to control axon termination
Source: PLoS Genet. 2022 Apr 14;18(4):e1010152. doi: 10.1371/journal.pgen.1010152 (PMC9041834; doi:10.1371/journal.pgen.1010152)
Supplement: S3 Table — (DOCX) [file pgen.1010152.s008.docx]

**Ubiquitin ligase activity inhibits Cdk5 to control axon termination**

Desbois *et al.*

**S3 Table CRISPR Targeting Sequences and Repair Templates**

| **Gene** | **crRNA** | **crRNA target sequence** | **Repair**  **Template** | **Repair Template Sequence** |
| --- | --- | --- | --- | --- |
| *rpm-1* | RPM-1 LD | CCATGTATTCGCCTCGGATG | RPM-1 LD ssODN | AAAGACTTGGAGCAGCACCATGTATTAGGCTGGGCGC***AGG***TGCATGTTCGCATTCCATTGTGTTCGAA TGATTTTGGAAAGAAGATG |
| *dpy-10* | *dpy-10* repair | GCTACCATAGGCACCACGAG | *dpy-10* ssODN | CACTTGAACTTCAATACGGCAAGATGAGAATGACTGGAAACCGTA***CCG***CATGCGGTGCCTATGGTAGCGGAGCTTCACATGGCTTCAGACC AACAGCCTAT |
| *cdk-5* | CDK-5 KD | TCGTTGCGTTGAAAAGAGTA | CDK-5 KD [K33T]  ssODN | GCCAGAAACAAAAATTCAGGAGAAATCGTTGCGTTGACCAGAGTA***CGT***TTGGACGATGATGATGAGGTACTCAAAAAGCATTCA |
| *cdk-5* | CDK-5 KD | AGCTGATTTTGGATTAGCCA | CDK-5 KD [D144N] ssODN | GCAAAACCTGCTGATTAATACAAATGGGACACTGAAGCTTGCTAATTTTGGATTAGCCA***GAG***CGTTTGGCGTCCCAGTTCGATGTTTTAGTGCAGAAG |
| *cdk-5* | CDK-5:: 3xFLAG | AAGTGTCTGCGAAGTACGCA | CDK-5:  :3xFLAG ssODN | GGAAGAATCGATGCCGATGCAGCTCTACG***TCA***TGCGTACTTCGCAGACACTTCTGACGTCGGAGGAGGAGGATCCGGAGGAGGAGGATCCGGAGGAGGAGGATCC ---3xFLAG --- CTATTCACTATTGCAC  TTTCCTACTC |
| *cdk-5* | CDK-5:: wrmScarlett | AAGTGTCTGCGAAGTACGCA | wrmScarlett ::CDK-5  PCR | GGAAGAATCGATGCCGATGCAGCTCTACG***TCA***TGCGTACTTCGCAGACACTTCTGACGTCGGAGGAGGAGGATCCGGAGGAGGAGGATCCGGAGGAGGAGGATCC ---wrmScarlett --- CTATTCACTATTGCAC  TTTCCTACTC |
| *fsn-1* | GFP::FSN-1 | AAGTATGGCTGAAAACGACG | GFP::FSN-1 PCR | GAAGCGAATGACAAGACTGTGGTGAGCTGGTTAATCGAGAAGT  --- GFP ---GGAGGAGGAGGATCCGGAGGAGGAGGATCCGGAGGAGGAGGATCCATGGCTGAAAACGACG***GTG***AAACGATTGTTCCAGACGAACAGTGCAATTTGACGGCCTC |

Legend: underline (crRNA targeting sequence), Italic bold (Pam sequence), Red (Insertion or gene edit), Blue (silent mutations in repair to prevent Cas9 re-cutting), Orange (linker)
