# Supplementary material for: Ubiquitin ligase activity inhibits Cdk5 to control axon termination
Source: PLoS Genet. 2022 Apr 14;18(4):e1010152. doi: 10.1371/journal.pgen.1010152 (PMC9041834; doi:10.1371/journal.pgen.1010152)
Supplement: S4 Table — (DOCX) [file pgen.1010152.s009.docx]

**Ubiquitin ligase activity inhibits Cdk5 to control axon termination**

Desbois *et al.*

**S4 Table Primers used for genotyping and cloning**

| **Gene** | **Allele/**  **construct** | **Primer sequence** |
| --- | --- | --- |
| *rpm-1* | *ju44* | ju44 fwd: 5' ttgttttccgttttatgcagtg 3’  ju44 rev: 5' cattaaacgctggaagtcatca 3’  digest with AccI |
| *rpm-1* | *bgg40 and bgg74* | rpm-1 ld fwd: 5 ‘agaagctctcaacggaatgg 3’  rpm-1 rev: 5’caggattgaactgcgagcta 3’ |
| *rpm-1* | *bgg6* | bgg6 fwd: 5’gatgagcagtggctggttag 3’  bgg6 int: 5’ tcgagaagcattgaacacca 3’  bgg6 rev: 5’ ctggtttccacgacttcacat 3’ |
| *fsn-1* | *bgg47* | bgg47 fwd: 5' ctgttccgtttcacatctccatc 3’  bgg47 int: 5' tcgagaagcattgaacacca 3’  bgg47 rev: 5' ccacggacaccgtctgttg 3’ |
| *fsn-1* | *gk429* | gk429 fwd: 5' ctgttccgtttcacatctccatc 3'  gk429 wt: 5’ ccacagtcttgtcattcgcttc 3'  gk429 rev: 5' cccaataccgttgtttcgtcc 3' |
| *cdk-5* | *ok626* | ok626 fwd: 5' ttacgtcacccgaacaaaca 3’  ok626 wt: 5' gagatctcaagccgcaaaac 3’  ok626 rev: 5’ tccggtatacgcaaatgaca 3’ |
| *cdk-5* | *bgg52* | bgg52 fwd: 5’gccctacccaatctaccatc 3’  bgg52 rev: 5’ctggactcggtttgacaatct 3’ |
| *cdk-5* | *bgg57* | bgg57 fwd: 5’acatggagccaaatcgtacc 3’  bgg57 int: 5’ acatgaattgtggggagagg 3’  bgg57 rev: 5’ tcattgtccaacaaaatctcg 3’ |
| *cdk-5* | *bgg71* | bgg71 fwd: 5’agatcttattctcccacgag 3’  bgg71 rev: 5’gagccttactacatttcggt 3’  digest with HpyCH4IV |
| *cdk-5* | *bgg77* | bgg77 fwd: 5’ accgaaatgtagtaaggctc 3’  bgg77 rev: 5’ ctggaagttgtgtaattgatggcc 3’  digest with HindIII |
| *cdka-1* | *tm648* | tm648 fwd: 5' ctaaatccgggtcatcgtcag 3’  tm648 wt: 5’ taccgtggcacatgtttc 3’  tm648 rev: 5’ggattatagtcttctttgtgcctc 3’ |
| *pct-1* | *tm2175* | tm2175 fwd: 5’ cagttgtcgacaagcttaac 3’  tm2175 wt: 5’ cattcgcgtgtcgaagattc 3’  tm2175 rev: 5’ gagtccaagttgctctgttg 3’ |
| *cdk-5* | genomic locus | MD148: 5’ gtagtccacttcaacgtgtcg 3’  MD151: 5’ gataacactttgataatttttaccttgtcattgtc 3’ |
| *cdk-5* | cDNA | MD96: 5’atgcttaactatgataaaatgg 3’  MD97: 5’ttagacgtcagaagtgtc 3’ |
| *cdk-5* | Kinase Dead point mutagenesis | MD170 (K33T): 5’ tcgtccaaccttactcttgtcaacgcaacgatttctc 3’  MD171 (K33T): 5’ gagaaatcgttgcgttgacaagagtaaggttggacga 3’  MD172 (D144N):5’ ccctggctaatccaaaattagctagtttcagtgtccc 3’  MD173 (D144N): 5’ gggacactgaaactagctaattttggattagccaggg 3’ |
| *cdka-1* | cDNA | MD174: 5’atgggcgcaaatttgacg 3’  MD175: 5’tcattcggaacttgaacaatgc 3’ |
