# Supplementary material for: Ubiquitin ligase activity inhibits Cdk5 to control axon termination
Source: PLoS Genet. 2022 Apr 14;18(4):e1010152. doi: 10.1371/journal.pgen.1010152 (PMC9041834; doi:10.1371/journal.pgen.1010152)
Supplement: Attachment — Submitted filename: Desbois et al, response reviewers_v2, final.docx [file pgen.1010152.s010.docx]

We would like to thank the reviewers for their time in evaluating our manuscript, and for noting the quality, novelty and importance of our study. The reviewers provided many valuable comments and suggestions. Based on their input, we have substantially revised our manuscript with numerous new experiments and textual adjustments. Below we summarize the major experimental revisions in our manuscript.

1) We demonstrate using CRISPR-based, native biochemistry that binding between the RPM-1 LD substrate ‘trap’ and CDK-5 is dependent on FSN-1 (**Fig 2G-I**). This provides more definitive evidence that CDK-5 shows substrate-like interactions with the RPM-1/FSN-1 ubiquitin ligase complex.
2) Our new results indicate that impairing RPM-1 catalytic ubiquitin ligase activity via CRISPR (*rpm-1 LD CRISPR*) results in ALM axon termination defects which are partially suppressed by a *cdk-5* null allele (**Fig 3B, E**). These outcomes mirror what occurred between *rpm-1* and *cdk-5* using null alleles (**Fig3B, C**). This provides further, more definitive genetic evidence that RPM-1 ubiquitin ligase activity regulates CDK-5 to control axon termination. This is important because several prior studies showed that RPM-1 utilizes both ubiquitin ligase activity and non-ligase signaling mechanisms to regulate axon termination.
3) We provide new results showing where CDK-5::mScarlet is localized in ALM neurons (**new Fig 6**). Our results indicate that CDK-5 was not detected in the axon but is present in the soma of ALM mechanosensory neurons. Moreover, we observe colocalization between CDK-5::mScarlet and the GFP::RPM-1 LD substrate ‘trap’ in the soma.
4) As requested by the editor and one reviewer, we evaluated how RPM-1 and CDK-5 affect synapse formation in ALM neurons. Our results indicate that RPM-1 and CDK-5 function in parallel to regulate synapse formation (**new Supplemental Fig 4**). Thus, RPM-1/FSN-1 inhibition of CDK-5 is required for proper termination of axon growth, but these molecules share a different functional relationship in the context of synapse formation. Based on these new findings, we have altered the title of manuscript to accurately reflect the overall conclusions of our study. We also comment on why this might be the case.
5) Previous studies showed that PCT-1 functions redundantly with CDK-5 to regulate formation of new synapses. We tested this possibility for axon termination. Our results indicate that PCT-1 does not function redundantly with CDK-5 to affect axon termination (**new** **Supplemental Figure 3**).
6) Finally, we exhaustively used cell-based assays with human proteins and *C. elegans* to see if we could detect effects of RPM-1/MYCBP2 on CDK5 ubiquitination. We also attempted ubiquitination experiments with RPM-1 and CDK-5 using *C. elegans*. Unfortunately, these experiments were inconclusive. Nonetheless, we think it is valuable for the reviewers and editor to know that we valued their suggestion and did all we could to try and address this.

Below are specific responses to all reviewer comments.

We hope the editor and reviewers agree that our revised paper in now suitable for publication in *PLoS Genetics*.

Reviewer #1: In this manuscript the authors' analyse their previous proteomics data and bring out the interaction between ligase dead RPM-1 and CDK-5. They confirm this interaction using in vivo biochemical studies using CRISPR-Cas9 strains. The authors’ further show a genetic interaction between cdk-5 and rpm-1 where cdk-5 mutants partially suppress the axon guidance defects in rpm-1 mutants in ALM and SAB neuron. The rescue experiments with ALM show that this is likely a cell autonomous rescue. The manuscript is well written and presented. However, I have multiple questions that relate specifically with the interpretation of the genetic experiments by the authors and the in vivo relevance of the interaction between CDK-5 and ligase dead RPM-1.

My comments are given below:

Major comments:

1. The authors show that CDK-5 interacts with Ligase Dead (LD) RPM-1, CDK-5 interacts with FSN-1 and the CDK-5, FSN-1 interaction is dependent on RPM-1 (LD). From the blots shown in figure 2 there is a clear indication that the RPM-1 (LD) version is what complexes with CDK-5, the blot also indicates that the WT RPM-1 does not interact with CDK-5. From his interaction it is unclear how CDK-5 is an RPM-1 substrate if the interaction is completely dependent on a ligase dead version of RPM-1. How does this translate in vivo and what is the relevance of this interaction in vivo. These points are not explained in the manuscript and are in my opinion very important points to address.

>>>

We agree with the reviewer that these important experiments and need to be presented more clearly. We have added substantial new text and cite our prior study (Crawley et al, Nature Communications, 2019) which showed that the RPM-1 LD acts as a substrate ‘trap’. By impairing RPM-1 RING ubiquitin ligase activity, substrates are no longer ubiquitinated but are still capable of binding to the FSN-1 substrate recognition module of the RPM-1/FSN-1 ubiquitin ligase complex. As a result, RPM-1 LD ‘traps’ and enriches substrate interactions with the RPM-1/FSN-1 complex. Substrate interactions with the wild-type RPM-1/FSN-1 ubiquitin ligase complex are presumably transient and harder to detect. We ask the reviewer to note that we have used the RPM-1 LD substrate ‘trap’ previously to identify UNC-51/ULK, which was validated as a conserved ubiquitination substrate that is degraded by the proteasome (Crawley et al, 2019).

We have addressed the reviewers concern in several ways:

i) We clarified our point with the following new text.

*“To enrich putative substrates, we relied upon an RPM-1 ubiquitin ligase dead (LD) construct that is catalytically inactive (****Fig 1A****). We previously demonstrated that the RPM-1 LD acts as a substrate ‘trap’ that enriches ubiquitination substrates without modifying them and triggering subsequent proteasome-mediated degradation (****Fig 1A****) [33].”*

ii) We add new quantitative experiments showing that endogenous CDK-5 binding to the RPM-1 LD substrate ‘trap’ is mediated by the FSN-1 substrate recognition module (**Fig 2G-I**). This result combined with our prior studies (Crawley et al, 2019) provides substantial evidence that the RPM-1 LD construct can ‘trap’ and enrich ubiquitination substrates.

iii) We would like to note that affinity purification proteomics with GS::RPM-1 and the GS::RPM-1 LD substrate ‘trap’ is done *in vivo*. Thus, we assume the reviewer is asking us to evaluate the functional effects of RPM-1 LD using genetic approaches in vivo. We agree that his is an excellent suggestion. Thus, we have used CRISPR editing to impair RPM-1 catalytic ubiquitin ligase activity and generated an *rpm-1 LD CRISPR* mutant. These animals display ALM axon termination defects (**Fig 3B, E**). Morevoer, we observe partial suppression in *cdk-5; rpm-1 LD CRISPR* double mutants. These results provide further, more definitive genetic evidence that RPM-1 ubiquitin ligase activity inhibits CDK-5 to control axon termination.

iv) We provide new data showing that the RPM-1 LD colocalizes with CDK-5 in the soma of ALM mechanosensory neurons (**new Fig 6**). This suggests that the RPM-1 LD substrate ‘trap’ spatially localizes in similar subcellular compartments as CDK-5. This result is consistent with our proteomic, biochemical and genetic findings, and further strengthens our study.

v) To further address the reviewer’s comment, we have adjusted our language to be less definitive about CDK-5 as an RPM-1 substrate. We now refer to “substrate-like interactions”

at several points in the Introduction, Results and Discussion.

2. I feel that the authors are overstating the conclusions from their genetic experiments. In the first set of genetic experiments performed using the length of the ALM axon, that authors show that WT and cdk-5 mutants show no phenotype, about 75% of rpm-1 mutants show a failed termination or overshooting of the axon phenotype and around 50% of the cdk-5; rpm-1 show the overshooting phenotype. This would imply partial suppression of the rpm-1 phenotype by cdk-5 mutants. I feel that concluding from this experiment that RPM-1 inhibits CDK-5 would be flawed. A similar problem is seen in case of fsn-1 mutants, where the experiments indicate that cdk-5 suppresses the fsn-1 mutant phenotype and does not show that FSN-1 inhibits CDK-5. Again, similar problems are seen with cdka-1; rpm-1 double mutants. In fact the suppression here seems to be fairly minimal, indicating only a partial suppression of the rpm-1 phenotype by cdka-1.

>>>

We understand the reviewer’s comment and appreciate the opportunity to clarify.

i) When using null alleles, a reduction in phenotype in a double mutant (genetic suppression) indicates that the mutant which has a reduced phenotype is upstream and acting in an inhibitory capacity. We can point the reviewer to several prior studies (by us and others) where similar conclusions are drawn from genetic suppression and were approved by numerous peer reviewers across a range of publication venues (Nakata… Jin, Cell 2005; Collins… Diantonio, Neuron 2006; Yan… Jin, Cell 2009; Baker… Grill, PLoS Genetics 2014; Baker… Grill, Genetics 2015; Borgen…Grill, Development 2017; Crawley…Grill, Nature Communications 2019).

ii) Nonetheless, we appreciate the reviewer’s potential point about clarifying partial suppression. While this does not invalidate our conclusion, it is worth discussing the potential reasons for why partial suppression might occur. First, redundancy with another gene/protein could prevent complete suppression. Second, if a gene functions through multiple downstream players or pathways partial suppression can be observed.

To address the first possibility (redundancy), we tested *pct-1* which was previously shown to function redundantly with CDK-5 during formation of new synapses (Park…Shen, Neuron, 2011). We did not observe suppression in *pct-1; rpm-1* double mutants compared to *rpm-1* single mutants (**Supplemental Fig 4**). Moreover, increased suppression was not observed in *cdk-5; pct-1;rpm-1* triple mutants compared to *cdk-5; rpm-1* double mutants (**Supplemental Fig 4**). These new results indicate that PCT-1 and CDK-5 do not function redundantly to affect axon termination. Our results do not rule out the possibility of another, unknown kinase still functioning redundantly with CDK-5.

The second point is actually known to be the case for RPM-1/MYCBP2 – it functions via multiple downstream players and pathways including through multiple downstream ubiquitination substrates. RPM-1 acts as a signaling hub that has several downstream effectors that control axon termination. These effectors are either substrates of its ubiquitin activity (UNC-51, DLK-1) or binding proteins that function with RPM-1 (GLO-4, PPM-2, RAE-1, ANC-1) (for review see Grill et al, Neural Development, 2016). The presence of multiple downstream effectors is another possible reason why we see partial suppression of *rpm-1* (lf) axon termination defects by *cdk-5*.

iii) Previous studies showed that RPM-1 and FSN-1 form a ubiquitin ligase complex that inhibits substrates via proteasome degradation (**Fig 1A**; Liao… Zhen, Nature 2004; Sharma…Grill, JBC 2014; Desbois…Grill, JBC 2018; Crawley… Grill, Nat Comm 2019; Nakata…Jin, Cell 2005; Yan… Jin, Cell 2009). Genetic suppression of *rpm-1* and *fsn-1* occurs with all other ubiquitination substrates that are degraded by the proteasome, such as UNC-51/ULK (Crawley… Grill, Nat Comm 2019) and DLK-1 (Nakata…Jin, Cell 2005; Yan… Jin, Cell 2009). Thus, suppression and an inhibitory genetic relationship would also occur if RPM-1/FSN-1 ubiquitination of CDK-5 resulted in proteasome-mediated degradation. We now provide commentary at multiple points in our revised paper to better explain this logic.

Results:

*Termination of axon growth has been used previously to evaluate the function of RPM-1*

*[42, 43], its binding proteins [37-39], and its ubiquitination substrates which are known to be degraded by the proteasome [33, 37, 44].*

Discussion:

*“To date, all known RPM-1 substrates are ubiquitinated and degraded by the 26S*

*proteasome [36, 44, 53-55].”*

*“These inhibitory genetic relationships [between cdk-5 and rpm-1] are similar to UNC-51/ULK and DLK-1 which are ubiquitinated by RPM-1 and targeted for proteasome-mediated degradation [33, 44]. While it remains unclear if RPM-1/FSN-1 inhibition of CDK-5 occurs via the proteasome, this is a reasonable possibility. However, our findings do not rule out the alternative of proteasome-independent inhibition.”*

iv) The partial suppression of *rpm-1* phenotype by *cdka-1* is explained by the fact that CDKA-1 is the upstream activator of CDK-5. If *cdka-1* is impaired, *cdk-5* activity will be reduced and suppression of *rpm-1* can occur in an *rpm-1; cdka-1* double mutant. This relationship between CDK-5 and CDKA-1/p35 is confirmed by the gain of function experiment we show in Figure 4C and B where overexpression of both CDK-5 and CDKA-1 are required to impair axon termination. For a similar example of this sort of genetic relationship, please see our genetic studies showing that the MIG-2 Rac functions as an upstream activator of DLK-1 (Borgen…Grill, Development 2017). Like *dlk-1*, *mig-2* loss of function is able to suppress *rpm-1* (lf) phenotypes. *mig-2* gain of function results in failed axon termination phenocopying *rpm-1* (lf).

v) Finally, we now include a new schematic summarizing our proteomic, biochemical and genetic findings as they pertain to axon termination (**new Fig 8**). Given the reviewer’s comments, we think this summary figure is likely to be valuable for readers.

3. The rescue experiments in figure 4 are useful in indicating that the suppression of rpm-1 is because of CDK-5 in mechanosensory neurons. The overexpression experiment indicates a possibility of CDK-5 inhibiting RPM-1 as the authors have stated, however it can be noted here that the overexpression phenotype of both CDK-5 and CDKA-1 together is only about 20% failed termination which is much less then what is seen in rpm-1 mutants, this would require a milder interpretation of the results seen. Further, the experiments in the manuscript allude to a complex interaction between CDK-5 and RPM-1 and parsing out this interaction may give a lot more insight into how the two molecules function.

>>>

Perhaps the reviewer has misstated their comment? We do not think that CDK-5 inhibits RPM-1. All our results support the opposite conclusion: that RPM-1 acts as an upstream inhibitor of CDK-5. Our overexpression results are consistent with this conclusion.

The less frequent phenotype observed with CDK-5 + CDKA-1 overexpression compared to *rpm-1* (lf) mutants is consistent with the partial suppressor effect we observe in *cdk-5; rpm-1* double mutants. A moderate suppressor effect would be expected to yield a moderate overexpression defect.

Once again, a mild-overexpression phenotype and a mild suppressor effect is not unreasonable given that RPM-1 has several downstream effectors that influence axon termination. More prominent players in RPM-1 signaling, such as DLK-1 and UNC-51/ULK, give stronger suppressor effects and stronger overexpression phenotypes (for examples see Baker… Grill, Genetics 2015; Crawley…Grill, Nature Communications, 2019; Yan and Jin, Cell 2009). Due to the length of our manuscript, we do not get into these details in the paper. However, if the reviewer thinks this is an important point, we could include this language.

4. The experiments in figure 5 are interpreted by the authors as RPM-1 specifically restricting the kinase activity of CDK-5, I think this would require showing a difference in CDK kinase activity in WT vs rpm-1 with more cells having kinase activity in rpm-1 mutants. I do agree that the experiments point to the fact that CDK-5 kinase activity is seen in ALM neurons for normal axon growth and termination.

>>>

We understand the reviewer’s comment.

We think that the combination of the *cdk-5 KD CRISPR* allele suppressing *rpm-1* (**Fig 5B**), transgenic rescue experiments showing CDK-5 kinase activity is required to rescue suppression in *cdk-5; rpm-1* double mutants (**Fig 5C**), and gain of function experiments with CDK-5/CDKA-1 overexpression (**Fig 4C, D**) indicate that CDK-5 and its kinase activity are required for proper axon termination.

Our conclusion from this series of observations is that RPM-1 inhibits CDK-5 kinase activity to regulate axon termination, rather than restricting some other unknown CDK-5 function that is kinase independent. While such kinase independent CDK-5 activity has not been described to our knowledge, we still chose to formally to test the role of CDK-5 kinase activity in axon termination. We consider these extensive results with transgenic and CRISPR edited animals to convincingly show that CDK-5 kinase activity is involved in axon termination and restrained by RPM-1.

While the reviewer’s suggestion that we biochemically test CDK-5 kinase activity is possible, we chose to focus extensive time and energy on a series of biochemical experiments attempting to more directly evaluate whether RPM-1 ubiquitination activity affects CDK-5. As such, we considered biochemical evaluation of CDK-5 kinase activity beyond the scope of this manuscript.

5. The SAB neuron experiment confirms the fact that cdk-5 partially suppresses the axon termination defects seen in rpm-1. This is a nice addition to the ALM experiments but again does not conclusively show that RPM-1 inhibits CDK-5 in this system.

>>>

We appreciate the reviewer noting the importance of showing that axon termination defects are suppressed in *cdk-5; rpm-1* double mutants in multiple types of neurons. We agree that this argues for the potential broad relevance of the genetic relationship between *rpm-1* and *cdk-5*.

Please see our extensive comments above explaining why genetic suppression with null alleles, genetic experiments showing *cdk-5* suppresses defects caused by an *rpm-1 LD CRISPR* allele, and gain of function experiments indicate that RPM-1 functions as an upstream inhibitor of CDK-5.

Minor comments:

1. The authors should indicate a list of primers in the supplemental tales detailing the primers used for making constructs and genotyping the strains used in this manuscript.

>>>

The reviewer’s point is well taken. We have added a new table (**Supplemental Table 4**) that shows all primers used for genotyping and cloning.

2. The plots indicating the proteomic data sets need to be better explained in the text or the figure legends. Plots B, D and D of figure 1 are difficult to understand and many terms like B ions and m/z are not described in the figure legends. Further, there is no methods section available for the proteomics experiment, this should be added to the methods. The authors do cite the method from their previous paper but a small section in this manuscript would help the reader.

>>>

We appreciate the reviewer pointing out the lack of details regarding proteomics.

To address this, we add further text to the legend of Figure 1: *“Shown are B ions formed from the amino terminus and Y ions formed from the carboxyl terminus. m/z represents mass to charge ratio with peptide size increasing to the right.”*

We agree that including a brief section in the Methods to explain proteomic procedures is useful for the reader. This is now included, see ***C. elegans proteomics*.**

3. Please describe the control CRISPR strains used (page 6, line 3).

>>>

Thank you for pointing this out. We now clarify the controls that were used in the experiment in the text of the Results.

*“We used CRISPR editing to generate rpm-1 LD CRISPR mutants, which were then crossed with animals carrying GFP::FSN-1 CRISPR and CDK-5::FLAG CRISPR. CoIP was performed with these animals, control CRISPR engineered strains expressing single constructs, and wild-type animals.”*

4. For the ALM axon guidance experiments please show both rpm-1 like and WT like phenotypes of cdk-5; rpm-1 animals. Currently, only the WT-like phenotype is indicated in panel 3A.
5. Similarly in panel 6A it may help the reader to show both phenotypes seen in SAB axons of cdk-5; rpm-1 C. elegans.

>>>

We appreciate the reviewer’s careful attention to detail and their request for more thorough presentation of phenotypes for *cdk-5; rpm-1* double mutants. We now include multiple images for *cdk-5; rpm-1* double mutants depicting both less severe axon termination defects and the presence of wild-type axon termination in ALM neurons (**Fig 3B**). Similar images are shown for the *cdk-5;* *rpm-1 LD CRISPR* double mutants, which are now included as part of our revised manuscript (**Fig 3B**). We make similar updates to our presentation of SAB phenotypes in *cdk-5; rpm-1* double mutants (**Fig. 7A**).

Reviewer #2: In this manuscript, Muriel Desbois et,. al uncover a surprising role of E3 ligase rpm-1 in regulating CDK-5. Both rpm-1 and cdk-1 have profound roles in neuronal development, and this study is the first to connect those two genes. Over all, the studies were well designed, and the proteomic and genetic data are convincing. I only have a few questions as following:

>>>

We thank the reviewer for highlighting the novelty and importance of our findings. We are also glad they appreciate the scope and value of combined proteomic and genetic approaches.

1) the only missing evidence is whether rpm-1 can directly degrade CDK-5 through UPS system. This can be tested by ubiquitin IP (or IP CDK-5 for ubiquitin ) in control and rpm-1(lf) animals. It will also help if the authors have data to show rpm-1(lf) increase CDK-5 protein levels in neurons.

>>>

The reviewer makes a valid point. We exhaustively attempted to do these experiments using both heterologous cell-based experiments with orthologous human proteins, and CRISPR tagged *C. elegans* strains. Unfortunately, our experiments were inconclusive. Nonetheless, we persevered and now provide several further *in vivo* experiments in *C. elegans* to address this comment.

i) We took our *in vivo* genetic experiments a substantial step further to provide more definitive evidence that RPM-1 ubiquitin ligase activity regulates CDK-5. To do so, we used CRISPR editing to generate *rpm-1 LD CRISPR* animals in which RPM-1 catalytic ubiquitin ligase activity is specifically impaired. Our results indicate that *rpm-1 LD CRISPR* mutants display axon termination defects in ALM neurons, and these defects are suppressed in *cdk-5;* *rpm-1 LD* *CRISPR* double mutants (**Fig 3B, E**). These results are now reported in a new subsection of our Results entitled: RPM-1 ubiquitin ligase activity inhibits CDK-5.

ii) We added a new native, CRISPR-based biochemical experiment from *C. elegans* suggested by another reviewer (see Reviewer 3’s comments). Our new quantitative biochemical results show that enriched binding of CDK-5 to RPM-1 LD is reduced in *fsn-1* (lf) mutants (**Fig 2G-I**). Thus, the RPM-1 LD substrate ‘trap’ enriches CDK-5 binding and this is mediated by the FSN-1 substrate recognition module. These results provide further evidence that CDK-5 displays substrate-like interactions with the RPM-1/FSN-1 ubiquitin ligase complex *in vivo*.

iii) We provide new data showing that RPM-1 LD colocalizes with CDK-5 in the soma of ALM neurons (**new Fig 6**). This suggests that the RPM-1 LD substrate ‘trap’ spatially localizes in similar subcellular compartments as CDK-5. This result is consistent with our proteomic, biochemical and genetic findings, and further strengthens our study.

iv) Finally, we have softened our language throughout the manuscript to be less definitive about CDK-5 as a substrate for the RPM-1/FSN-1 ubiquitin ligase complex. We now refer to “substrate-like interactions”.

2) as both rpm-1 and cdk-1 are involved in synapse formation. It will be interesting to test whether CDK-5 also functions downstream of rpm-1 in synapse formation. This should be straight forward by testing the suppression ability of cdk-5(lf) on rpm-1(lf) synapse phenotype.

>>>

The reviewer makes a valuable suggestion. The reviewer is correct in noting that RPM-1 has multiple roles in neuronal development. We agree that expanding our experiments in the direction suggested would make for a more comprehensive study. There is further value in this experimental suggestion. To our knowledge, only one prior study (Schaeffer… Nonet, Neuron 2000) has shown that RPM-1 regulates synapse formation in ALM neurons.

We used a combination of transgenes that express RFP and the presynaptic marker RAB-3::GFP to evaluate synapse formation in ALM mechanosensory neurons. Our results indicate that *rpm-1* mutants have a significant, but mild defect in ALM synapse formation (**new Supplemental Figure 4**). Having reproduced prior results, we evaluated ALM synapse formation in *cdk-5* single mutants and *cdk-5; rpm-1* double mutants. Our results indicate that *cdk-5* single mutants do not have defects in ALM synapse formation (**Supplemental Figure 4B**). *cdk-5;rpm-1* double mutants showed enhanced synaptic defects compared to *rpm-1* single mutants (**Supplemental Figure 4B**). Thus, RPM-1 and CDK-5 potentially function in parallel pathways to regulate synapse formation. This observation is reasonable as prior studies have shown that RPM-1 and CDK-5 affect different steps in synapse formation. RPM-1 regulates developmental synapse maintenance the last step in synapse formation (Borgen…Grill, eLife 2019). CDK-5 regulates synapse formation and is likely to affect synapse assembly an early step in synapse formation (Park…Shen, Neuron 2011).

We have added a new subsection in the Results to present this data entitled: *“RPM-1 and CDK-5 act in parallel to control synapse formation”*. We keep the overall focus of our paper and conclusions on RPM-1/FSN-1 inhibition of CDK-5 in the context of axon termination. This is because our experiments regarding axon termination are far more extensive and conclusive. Further experiments (e.g. transgenic rescue and CRISPR allele data) would be needed to definitively establish that RPM-1 and CDK-5 function in parallel to regulate ALM synapse formation.

To incorporate these new findings and ensure the accuracy of our study, we have now adjusted our title to the following: *Ubiquitin ligase activity inhibits Cdk5 to control axon termination*. We have also modified the abstract to note the functional context, axon termination, where the RPM-1/FSN-1 ubiquitin ligase inhibits CDK-5.

Reviewer #3: In this manuscript, Desbois et al showed that CDK5 is preferentially pull down by the kinase-dead RPM-1 but not by the wild type RPM-1 in worms. Biochemical analysis showed that RPM-1 interacts with CDK5, probably through FSN-1. Genetic analysis demonstrated that cdk5 loss of function rescues rmp-1 mutant phenotype, while gain of function of CDK5 mimics rpm-1 loss of function. These genetic analyses provided the most compelling evidence supporting the author’s argument and also suggested that the kinase activity of CDK5 is key in the RPM-1-mediated restriction of CDK5 activity in neurons. This study provided a potential novel mechanism in restraining CDK5 activity through the ubiquitin-mediated pathway.
I do have the following concerns:

>>>

We appreciate the reviewer noting the novelty of our findings and value of our experiments spanning proteomic, biochemical and genetic approaches.

Major:
1) One important outcome of the RPM-1-mediated regulation of CDK5 is not addressed (or even discussed): A] does the RPM-1/FSN-1 ubiquitin ligase targets CDK5 via polyubiquitination, which in turn promotes the proteasome-mediated degradation of CDK5? The data that in RPM-1 LD background, the interaction between FSN-1 and CDK5 is much stronger than in the wild type background seem to suggest that. However, easy experiments directly asking whether CDK5 abundance is elevated in rpm-1 or fsn-1 loss of function should be performed to clarify the issue and provide more solid and direct evidence to support the authors’ conclusion. The CRISPR allele CDK5-FLAG could be used if an endogenous CDK5 antibody is not available. In addition, whether the CDK5 kinase activity is enhanced in rpm-1 or fsn-1 mutants could also be tested. B] Another less-likely possibility is that RPM-1/FSN-1 mediates mono-ubiquitination of CDK5, which in turn suppress CDK5 kinase activity. Again, biochemical analysis of CDK5 abundance and SDS-PAGE migration pattern (in conjunction with Ubiquitin western blots) would provide a cleaner picture.

>>>

The reviewer makes several valuable points which we address in below.

We exhaustively attempted to do ubiquitination experiments with RPM-1 and CDK-5 using both heterologous cell-based experiments with orthologous human proteins, and CRISPR tagged *C. elegans* strains. Our efforts on these experiments, rather than being easy, took months to optimize and test. Unfortunately, these experiments were inconclusive. Nonetheless, we persevered and now provide several further *in vivo* experiments in *C. elegans* to address this comment.

We used CRISPR editing to specifically inactivate RPM-1 catalytic ubiquitin ligase activity and generated *rpm-1 LD CRISPR* animals. Our new results indicate that *rpm-1 LD CRISPR* mutants display axon termination defects in ALM neurons (**Fig 3B, E**). More importantly, axon termination defects are significantly suppressed in *cdk-5;* *rpm-1 LD* *CRISPR* double mutants compared to *rpm-1 LD CRISPR* single mutants (**Fig 3B, E**). These findings provide further support for our conclusion that RPM-1 ubiquitin ligase activity inhibits CDK-5 to control axon termination.

As suggested by the reviewer, we also examined if the levels of CDK-5 change in *rpm-1* (lf) mutants. To do so, we generated an endogenous CDK-5::mScarlet reagent via CRISPR engineering. This new reagent was necessary, as anti-FLAG antibodies do not work well for immunostaining in *C. elegans*. We examined CDK-5::mScarlet CRISPR levels in *rpm-1* mutants. As we discuss further below, CDK-5::mScarlet was observed in the ALM soma. Unfortunately, we did not observe significant changes in CDK-5 levels in *rpm-1* mutants. This does not invalidate any of our proteomic, biochemical or genetic findings. Our inability to detect changes in CDK-5 levels in *rpm-1* mutants might occur for several reasons.

i) Other RPM-1 substrates that are known to be polyubiquitinated and degraded by the

proteasome include UNC-51/ULK (Crawley…Grill, Nat Communs 2019) and DLK-1

(Nakata…Jin, Cell 2005). In the case of DLK-1 and UNC-51, very little or no protein is

detected in the adult nervous system of *C. elegans* creating high sensitivity for detecting

changes in protein levels. As a result, it is possible to observe changes in levels of DLK-1

and UNC-51 in *rpm-1* mutants. In contrast, we observe CDK-5::mScarlet CRISPR in

non-neuronal tissue and at relatively high levels across the *C. elegans* nervous system of

adult animals. This reduces sensitivity and potentially affects our ability to detect changes

in CDK-5 levels.

ii) CDK-5 turnover could be very slow or only happen to a small amount of CDK-5. For

example, there might be a relatively small pool of activated CDK-5 compared to the total

pool of CDK-5. We have no way to differentiate activated CDK-5 in *C. elegans* using our

reagents to test this second quite reasonable possibility. Our cell-based experiments (not

included in revision) did suggest that CDK-5 turnover is extremely slow. In contrast, the

MYCBP2 (RPM-1 human ortholog) substrate NMNAT2 is turned over in a matter of

minutes in cell-based assays (Desbois..Grill, JBC 2018; Babetto… Diantonio, Cell

Reports 2013). This facilitates extremely robust assays for ubiquitination and protein

level changes. This was not the case with human CDK5 in similar experiments we

attempted.

iii) It is possible that multiple ubiquitin ligases influence CDK-5 degradation. We note

this possibility in our Discussion. However, we point out that other ubiquitin ligases have

only been evaluated in cell-based assays using non-neuronal cells to date. Examining the

potential interplay between different ubiquitin ligases on CDK-5 levels and stability is

beyond the scope of this initial study, where we provide the first evidence that ubiquitin

ligase activity regulates CDK-5 *in vivo* and in the nervous system.

iv) To further address the reviewer’s comment, we have softened our language throughout the manuscript to be less definitive about CDK-5 as a substrate for the RPM-1/FSN-1 ubiquitin ligase complex. We now refer to “substrate-like interactions”.

v) Finally, the reviewer makes a reasonable point about RPM-1/FSN-1 ubiquitination of

CDK-5 and proteasome-mediated degradation. We appreciate the reviewer noting this.

This is a reasonable model, given prior studies have shown that all known RPM-1 substrates are ubiquitinated and degraded by the proteasome. At several points in the Results and Discussion, we now note that this is the most likely explanation for how RPM-1/FSN-1 ubiquitin ligase activity restrains CDK-5.

Results:

*“Termination of axon growth has been used previously to evaluate the function of RPM-1 [42, 43], its binding proteins [37-39], and its ubiquitination substrates which are known to be degraded by the proteasome [33, 37, 44].*

Discussion:

*“To date, all known RPM-1 substrates are ubiquitinated and degraded by the 26S proteasome [36, 44, 53-55].”*

*“These inhibitory genetic relationships [between cdk-5 and rpm-1] are similar to UNC-51/ULK and DLK-1 which are ubiquitinated by RPM-1 and targeted for proteasome-mediated degradation [33, 44]. While it remains unclear if RPM-1/FSN-1 inhibition of CDK-5 occurs via the proteasome, this is a reasonable possibility. However, our findings do not rule out the alternative of proteasome-independent inhibition.”*

2) In addition to the biochemical IP experiments, imaging analysis of co-localization of FLAG-CDK5 with GFP-RPM-1 (WT or DL) or GFP-FSN-1 in SAB motorneurons would provide more evidence supporting the physical interaction.

>>>

The reviewer makes an excellent suggestion.

We performed this experiment by using CRISPR to engineer an mScarlet tag onto endogenous CDK-5 (CDK-5::mScarlet CRISPR). Our new results indicate that CDK-5::mScarlet CRISPR is localized to puncta in the soma of ALM mechanosensory neurons (**new Fig 6B**). We used a cell-specific GFP transgene for a portion of our experiments to ensure we are accurately visualizing ALM neurons (**Fig 6B, D**). We further combined CDK-5::mScarlet CRISPR with transgenic GFP::RPM-1 LD expressed specifically in ALM mechanosensory neurons (**Fig 6C, E**). Our results show that RPM-1 LD colocalizes with CDK-5 in the ALM soma (**Fig 6C**). We provide further data showing that while GFP::RPM-1 LD is also concentrated at the ALM axon termination site, CDK-5::mScarlet is not present in this location (**Fig 6E**). Thus, the RPM-1 LD substrate ‘trap’ spatially localizes to similar subcellular compartments as CDK-5 in the soma of ALM neurons. These imaging results are consistent with our proteomic, biochemical and genetic outcomes. We present these findings as a new subsection of the Results: *CDK-5 and RPM-1 colocalize in the neuronal soma*.

Our observation that RPM-1 LD colocalizes with CDK-5 in the ALM soma suggests that RPM-1/CDK-5 signaling in the cell body might influence cellular events at the distal axon termination site. This is not an unprecedented concept as our prior work showed that RPM-1 binds and functions with the nuclear anchoring protein ANC-1/Nesprin (Tulgren…Grill, PLoS Genetics 2014). We note this point with the following new text in the Discussion:

*“Consistent with these results, we found that CDK-5 colocalizes with the RPM-1 LD substrate ‘trap’ in the soma of ALM mechanosensory neurons (****Fig 6****). Thus, our results suggest that RPM-1 might inhibit CDK-5 in the soma to regulate axon termination at a distal site (****Fig 8****). Our findings are not the first example of RPM-1 affecting signaling in the cell body. RPM-1 was previously shown to interact with a nuclear anchoring protein, ANC-1/Nesprin, to affect axon termination [72].”*

3) A key point brought up by the authors is that FSN-1 bridges the interaction between RPM-1 and CDK5. It would be interesting to test whether RPM-1 LD could pull down CDK5 in the fsn-1 null mutant background.

>>>

The reviewer again makes a thoughtful, valuable suggestion. Outcomes from such an experiment will strengthen our conclusion that the RPM-1/FSN-1 ubiquitin ligase complex regulates CDK-5. It would also provide more insight into the biochemical mechanism underpinning CDK-5 interactions with the RPM-1/FSN-1 ubiquitin ligase complex. Finally, it would further solidify the conclusion that CDK-5 displays substrate-like interactions with the RPM-1/FSN-1 ubiquitin ligase complex.

Building upon the reviewer’s insightful suggestion, we have now added a new native, CRISPR-based biochemical experiment from *C. elegans*. Quantitative results indicate that enriched CDK-5 binding to RPM-1 LD is reduced in *fsn-1* (lf) mutants (**Fig 2G-I**). Thus, the RPM-1 LD substrate ‘trap’ enriches CDK-5 binding via the FSN-1 substrate recognition module.

We would like to thank the reviewer. Their suggestion has pushed us beyond the limit of what we would have considered attempting with native, CRISPR-based biochemistry in *C. elegans*.

Minor:
I would advise against using the term “in vivo, native biochemistry”. The analysis is completely in vitro when you use detergent to dissolve all the cellular and intracellular boundaries, in order to generate the cell lysate. The only difference from a typical CO-IP experiment is that, instead of using antibodies to IP the endogenous proteins, you generated endogenous epitope tags, which does not make the biochemical analysis in vivo.

>>>

We beg to differ with the reviewer about this terminology. We are extracting proteins and complexes for a whole animal setting. Thus, we consider these “in vivo” biochemical experiments. When we overexpress multiple tagged *C. elegans* or human protein components in cell lines and perform biochemistry, we refer to this as “heterologous expression” or “cell-based” biochemistry. If biochemistry is done with individual protein components purified to homogeneity from *E. coli* or cells, we would use the term “in vitro”.

Nonetheless, we respect the reviewer’s opinion and have opted for a compromise in our revised manuscript. We primarily use the term “native CRISPR-based biochemistry” as much as possible. However, at times, we still note the “in vivo” nature of our experiments.

As a final consideration, we point the reviewer to several published papers where other reviewers have allowed us to use the term “in vivo” to describe proteomics and biochemistry done from *C. elegans* (Grill… Jin, Neuron 2007; Grill…Garner, J Neurosci, 2012; Baker…Grill, PLoS Genetics 2014; Tulgren… Grill, PLoS Genetics 2014; Crawley… Grill, Nature Communications 2019; Giles…Grill, JBC 2019).
